# Capillary effect-based selective sealing strategy for increasing piezoelectric MEMS speaker performance

**DOI:** 10.1038/s41378-024-00753-x

**Published:** 2024-08-07

**Authors:** Yan Wang, Tunan Lv, Junning Zhang, Hongbin Yu

**Affiliations:** 1 School of Optical and Electronic Information, Huazhong University of Science and Technology, Wuhan, 430074 China; 2 Optics Valley Laboratory, Wuhan, Hubei 430074 China; 3 Wuhan National Laboratory for Optoelectronics, Huazhong University of Science and Technology, Wuhan, 430074 China

**Keywords:** Engineering, Physics

## Abstract

To address the serious acoustic performance deterioration induced by air leakage in the low-frequency range and the asynchronous vibration in electroacoustic transduction structures near the resonant frequency, a novel sealing strategy is proposed that targets one of the most widely reported piezoelectric MEMS speaker designs. This design consists of multiple cantilever beams, in which the air gaps between cantilevers are automatically and selectively filled with liquid polydimethylsiloxane (PDMS) via the capillary effect, followed by curing. In the proof-of-concept demonstration, the sound pressure level (SPL) within the frequency range lower than 100 Hz markedly increased after sealing in an experiment using an IEC ear simulator. Specifically, the SPL is increased by 4.9 dB at 20 Hz for a 40 V_pp_ driving voltage. Moreover, the deteriorated SPL response near the resonant frequencies of the cantilever beams (18 kHz–19 kHz) caused by their asynchronous vibration induced by the fabrication process nonuniformity also significantly improved, which successfully increased the SPL to approximately 17.5 dB. Moreover, sealed devices feature nearly the same SPL response as the initial counterpart in the frequency band from 100 Hz to 16 kHz and a total harmonic distortion (THD) of 0.728% at 1 kHz for a 40 V_pp_ driving voltage. Compared with existing sealing methods, the current approach offers easy operation, low damage risk, excellent repeatability/reliability and excellent robustness advantages and provides a promising technical solution for MEMS acoustic devices.

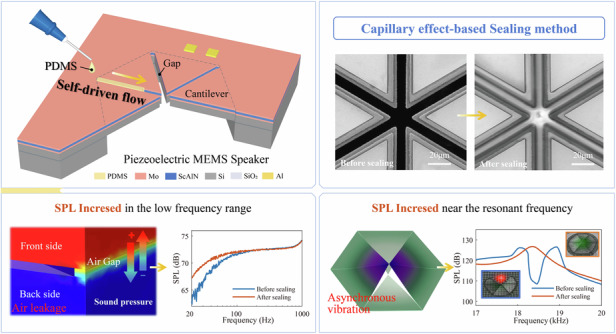

## Introduction

With the development of the Internet of Things and wearable electronic devices, demand for low power consumption, miniaturization and intelligent mobile devices is growing^1^. Traditional electromagnetic speakers face technical challenges because of their large size, high power consumption and complex manual assembly^2^. Thus, these speakers do not easily adapt to the wave of intelligent device development. Therefore, MEMS speaker technology has received significant attention in recent years, and different types of MEMS speakers have been proposed, including electromagnetic^3,4^, electrostatic^5,6^ and piezoelectric speakers^7–12^. Among them, piezoelectric MEMS speakers have been treated as the most promising substitute because of their relatively simple structure, high operation reliability, low power consumption and moderate performance^13–17^. To date, great progress has been made in this area, and various piezoelectric MEMS speakers have been developed for high-frequency^7^, broadband^8^ and directional emission^9^ applications. Based on electro-acoustic transduction structure design, piezoelectric MEMS speakers can be roughly categorized into three groups^1^, namely, fully clamped diaphragms^10^, partially clamped diaphragms^11–14^ and cantilever beam-like actuators^7,8,15,16^. Traditional fully clamped diaphragm structures suffer from problems such as high stiffness, low displacement sensitivity and poor linearity. To achieve good linearity and stress insensitivity, researchers have developed cantilever beam-like actuator structures formed by etching air gaps into a fully clamped diaphragm. The cantilever beam-like actuator design refers to a vibrating membrane structure consisting of one or more cantilever beams that are fixed at one end and free at the other. Considering the structural characteristics, the cantilever beam possesses greater deformation capability and can release residual stresses in the as-deposited materials via deformation. Due to the associated high SPL output capability, large linear operation range and low sensitivity to fabrication process-related residual stress advantages, cantilever beams, such as actuator designs, have attracted increasing amounts of extensive study.

Unfortunately, the air gaps surrounding cantilever beams can provide an air leakage pathway, causing “acoustic short circuit” issues. The air on both sides of the diaphragm can be exchanged through the gaps, resulting in a decrease in the low-frequency SPL of the speaker^12,13^. In addition, the speaker usually consists of multiple cantilever beams, each of which can operate independently without any mechanical coupling. Because the resonant frequency depends on the size of the cantilever beams, fabrication dimension deviations can lead to inconsistent resonant frequencies between the cantilever beams. During speaker operation, when the driving frequency exceeds the resonant frequency of the cantilever beam, the phase of the cantilever beam will reverse. As the resonant frequencies of different cantilever beams are inconsistent, the frequencies at which phase reversal occurs also differ, leading to a well-known asynchronous vibration phenomenon^7,8^. As a result, the sound signals emitted from these cantilever beams cancel, significantly decreasing the output SPL near the resonant frequency.

A narrower air gap and more rigorous process control can be used to address the air leakage problem at the expense of distinctly increased difficulty. Moreover, both the residual stress-induced initial structural deformation and the relatively large vibration amplitude during operation can inevitably introduce additional gap widening, and the problem thus remains unsolved. In fact, sealing the air gap is the most fundamental solution to solve the air leakage problem. Additionally, because the sealing material acts as a connecting spring between separate beams, asynchronous vibration phenomena can also be suppressed. Sealing techniques can be applied before or after the release of the diaphragm, with sealing materials such as PDMS and Parylene-C^18–21^. In the work of Ref. ^18^ and Ref. ^19^, a sealing method involving the deposition of Parylene-C before release was used. However, because Parylene-C is deposited on the diaphragm surface before release, surface Parylene-C inhibits the deformation of the cantilever beam, affecting the release of residual stress. Inadequate release of residual stress can lead to increased structural stiffness and decreased sensitivity of the speaker. In addition, the deposition of Parylene-C requires specialized equipment and increases the complexity of subsequent processes. Finally, the high Young’s modulus of Parylene-C increases the structural stiffness, thus changing the original device performance accordingly.

In the work of Ref. ^20^, the entire surface of the MEMS speaker was covered by a 15 μm thick polydimethylsiloxane (PDMS) film after release to block air leakage, which resulted in a 15 dB SPL improvement at 20 Hz. Due to postprocessing and the low Young’s modulus of PDMS, this method does not require changes to the process flow, does not affect stress release in the cantilever beams, and has minimal impact on the original mechanical performance of the speaker. Nevertheless, postrelease cantilever beams are relatively fragile and usually demonstrate warpage, making uniform and reliable film coverage difficult and introducing risks of structural damage. Additionally, precise manual operation is needed for film coverage, making the experimental process difficult to control. Furthermore, the reliability and repeatability of film coverage and sealing operations are also difficult to guarantee. Kuchiji H.^21^ also employed a postprocessing method, but the coated polyurethane material had a relatively high Young’s modulus, resulting in the degradation of the performance of the device in the high-frequency range.

To explore a better solution, a novel postrelease sealing strategy for the MEMS speaker is presented in this paper that utilizes capillarity force, which is a common driving mechanism that moves fluids within capillary channels without requiring the use of costly and/or bulky pumps or other actuation systems in microfluidic systems^22^. In the current case, liquid PDMS is used to selectively fill the air gaps between cantilevers via the capillary effect, followed by curing. Because filling is a self-driven process, the implementation and process control can be significantly simplified, and high reliability and repeatability can be easily achieved. Moreover, the capillary effect only occurs in the air gap region, which permits a highly constrained PDMS distribution. Combining the low Young’s modulus and excellent elasticity of cured PDMS, sealing has a negligible effect on the mechanical performance of the existing speaker structure itself.

## Materials and methods

### Design and theoretical analysis

**Fig. 1:**
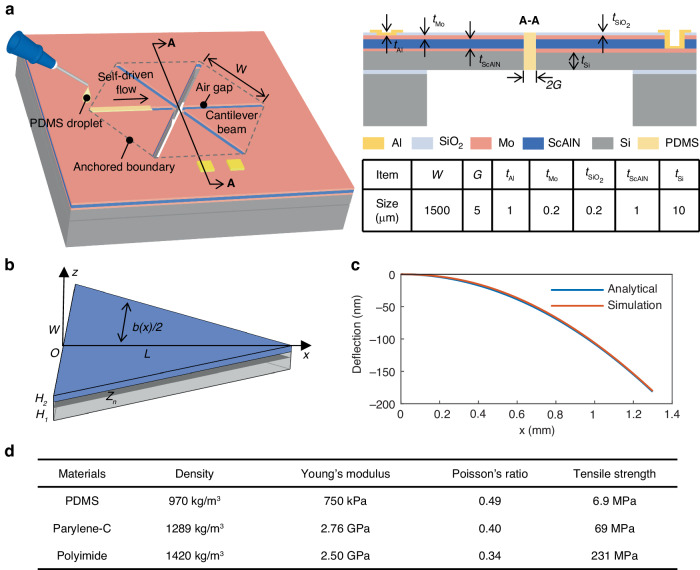
**Design concept of piezoelectric MEMS speaker. a** Illustration of the proposed piezoelectric MEMS speaker with sealing strategy. **b** Schematic of a triangular cantilever. **c** The z-direction deflection of the cantilever along the longitudinal direction. **d** Properties of common polymer materials^35,36^

The piezoelectric MEMS speaker used in the current proof-of-concept demonstration consists of six identical triangular composite cantilever beams, which act as the electroacoustic transduction structure. These cantilever beams are constructed from a Mo (top electrode)-ScAlN (piezoelectric layer)-Mo (bottom electrode) sandwich-like piezoelectric actuator structure deposited onto a silicon support layer. A 9.6% Sc doping concentration is utilized to enhance the piezoelectric driving capability of AlN^23,24^. The cantilever beams are separated by air gaps and arranged into a hexagon with their fixed ends anchored to the SOI substrate, as shown in Fig. 1a. The electrodes of the six cantilever beams are connected in parallel by the outer electrodes on the substrate region.

In this design, PDMS is used as the sealing material. The properties of PDMS and other common polymer materials are provided in Fig. 1d. PDMS has the lowest Young’s modulus among these materials and is only applied to the air gaps between cantilever beams, thus minimizing its impact on the mechanical characteristics of the structure as much as possible. The process of gap filling via the capillary effect is also schematically illustrated in Fig. 1a. Liquid PDMS droplets are placed on the end edge of the air gaps. Because the gaps of MEMS speakers can be regarded as suspended channels, as stated in Ref. ^25^, liquid PDMS is automatically drawn into narrow air gaps as a result of surface tension. The PDMS flows into the device to effectively and selectively fill the gaps, and this process inherently stops when equilibrium is reached. This sealing method is easy to perform and does not require strict control of the amount of applied PDMS. By repeating the above operation, all of the air gaps in the speaker can be sequentially filled. Afterward, the device is placed into an oven to cure the PDMS. The detailed structural parameters used in the current proof-of-concept demonstration are also provided in Fig. 1a.

Figure 1b shows a triangular cantilever beam anchored at the base with length and width denoted as *L* and *W*, respectively. The thicknesses of the supporting layer and the piezoelectric layer are denoted as ${H}_{1}$ and ${H}_{2}$, respectively. The position of the neutral plane of the double-layer cantilever is ${Z}_{n}$. Assuming that the base of the cantilever beam is at the coordinate origin, the cross-sectional width of the triangular cantilever beam gradually decreases from the base to the tip:

1 $$b\left(x\right)=W\frac{L-x}{L}$$

When a driving voltage, *U*, is applied between the top electrode and the bottom electrode, the resultant bending moment, $M\left(x\right)$, can be expressed as follows:

2 $$M\left(x\right)=-{e}_{31}{Ub}(x){h}_{e}$$

where $M\left(x\right)$ represents the bending moment at coordinate $x$ and ${e}_{31}$ is the piezoelectric coefficient. ${h}_{e}$ is the distance between the midplane of the piezoelectric layer and the neutral plane, expressed as follows^26^:

3 $${h}_{e}=\frac{{H}_{2}}{2}+{H}_{1}-{Z}_{n}$$

4 $${Z}_{n}=\frac{\sum _{i}{E}_{i}{y}_{i}{H}_{i}}{\sum _{i}{E}_{i}{H}_{i}}$$

where ${E}_{i}$ and ${y}_{i}$ represent Young’s modulus and the position of the midplane of the ith layer, respectively.

The upper and lower thin films have the same curvature radius and satisfy the following:

5 $$R=\frac{{d}^{2}w}{d{x}^{2}}=-\frac{M(x)}{{EI}(x)}$$

where the bending stiffness of the multilayer plate, ${EI}(x)$, and the moment of inertia of each layer, ${I}_{i}\left(x\right)$, are defined as follows^27^:

6 $${EI}\left(x\right)=\sum _{i}{E}_{i}{I}_{i}\left(x\right)$$

7 $${I}_{i}(x)=\frac{{{t}_{i}}^{3}+12{t}_{i}{\left({y}_{i}-{Z}_{n}\right)}^{2}}{12}b\left(x\right)$$

Substituting Eq. (6) and Eq. (7) into Eq. (5) yields the following:

8 $$\frac{{d}^{2}w}{d{x}^{2}}=-\frac{12M(x)}{b(x)K}$$

9 $$K=\sum _{i}{E}_{i}({{t}_{i}}^{3}+12{t}_{i}{\left({y}_{i}-{Z}_{n}\right)}^{2})$$

The boundary conditions of the single-end fixed cantilever beam satisfy the following:

10 $$\left. \frac{dw}{dx}\right|_{x=0}=0$$

11 $$w{{\rm{|}}}_{x=0}=0$$

By taking the second integral of Eq. (5) and combining Eq. (10) and Eq. (11), the deflection expression of the triangular cantilever under voltage, $U$, can be obtained:

12 $$w\left(x\right)=\frac{6{e}_{31}{h}_{e}U}{K}{x}^{2}$$

The deflection curve of the designed cantilever actuator along the length direction for a voltage drive of U = 1 V is shown in Fig. 1c. The theoretical and simulation results agree well.

The first-order resonant frequency of the triangular tapered cantilever is given by the following^28^:

13 $${f}_{{\!tri}}=\frac{0.659}{{L}^{2}}\sqrt{\frac{{E}_{1}{\left({H}_{1}-{Z}_{n}\right)}^{3}+{E}_{1}{{Z}_{n}}^{3}+{E}_{2}{\left({H}_{1}+{H}_{2}-{Z}_{n}\right)}^{3}-{E}_{2}{\left({H}_{1}-{Z}_{n}\right)}^{3}}{{\rho }_{1}{H}_{1}+{\rho }_{2}{H}_{2}}}$$

where ${\rho }_{1}$ and ${\rho }_{2}$ represent the densities of the two-layer materials.

Equation (13) shows that the resonant frequency of the triangular cantilever beam structure is inversely proportional to the square of its length. Considering the currently adopted cantilever design with a width of 1500 μm and a length of approximately 1299 μm, the resonant frequency of the cantilever beam is clearly sensitive to its length; a variation of 50 μm in length results in a frequency shift of 1.42 kHz. This sensitivity implies that any nonuniformity in the back cavity etching during the manufacturing process can easily lead to different lengths in the six cantilever beams, resulting in an inconsistency in their resonant frequencies. Because a well-known phase reversal characteristic will occur for a structure operating around its resonant frequency when all of the cantilever beams are driven with the same signal, the discrepancy in the resonant frequency will induce asynchronous vibration between the beams. As a result, the sound signals generated by these cantilever beams cancel each other, introducing a remarkable drop in the SPL frequency spectrum^7^.

### Numerical simulation

**Fig. 2:**
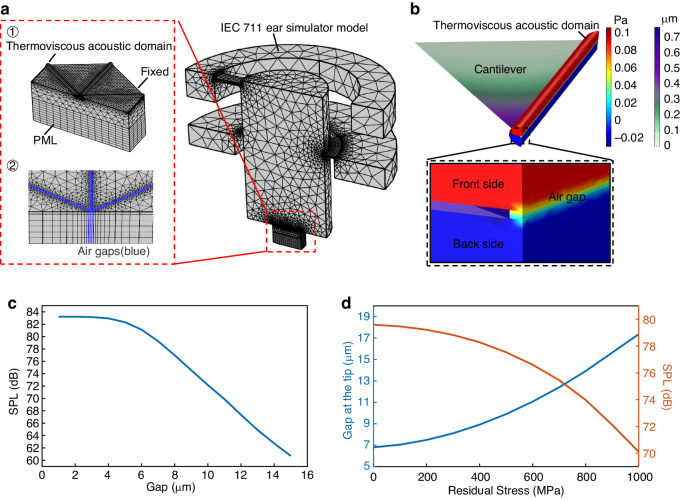
**Simulation results of piezoelectric MEMS speaker. a** Finite element simulation model and mesh partition. **b** Sound pressure distribution near the air gap. **c** Relationship between SPL and gap at 20 Hz. **d** The effect of the residual stress on the air gap between the cantilever tips and the resultant SPL at 20 Hz

A simulation model of the proposed piezoelectric MEMS speaker using the IEC 711 ear simulator available in the COMSOL Multiphysics library was established, as shown in Fig. 2a^29^. In the simulation, 1/2 symmetric processing is employed to simplify the model, reduce the computational burden in 3D finite element simulations and ease memory demands. The MEMS speaker is represented by considering only two layers, the piezoelectric layer and the support layer. A “fixed constraint” boundary condition is applied at the base of the cantilever beams. To simulate the open back cavity, the backside of the speaker is placed in a free sound field environment with multiple perfectly matched layers (PMLs) to absorb the sound pressure.

To account for the frictional losses caused by air within the gaps and accurately estimate the acoustic losses from both sides of the cantilever beam through the gaps, the air gaps and their adjacent regions (cylindrical domain) are defined as the “thermoviscous acoustic” domain. Based on the model described above, a transient simulation was conducted to calculate the sound pressure distribution on both sides of the cantilever beam, as illustrated in Fig. 2b. For clarity, only one cantilever and one air gap are provided in the figure to disclose the results from the thermoviscous acoustic domain near the air gap. When the cantilever is driven to vibrate, opposite sound pressures are excited on its front and back sides, and they are connected through the air gap, resulting in acoustic short-circuit phenomena and inducing acoustic loss. Moreover, the pressure sound field characteristics within the ear simulator permit a uniform sound pressure distribution^3,29^.

The SPL generated at 20 Hz for a 40 V_pp_ driving voltage by the proposed speaker before sealing was studied as a function of the air gap width, as shown in Fig. 2c. The acoustic loss-induced SPL reduction is negligible for air gap widths of less than 5 μm, as reported by others^14^. Wider air gaps result in a distinct decrease in the SPL. According to the following experimental results, the chlorine-based dry etching process for ScAlN increases the air gap width from the initially designed width of 5 μm to 6.8 μm, which results in a nearly 2.66 dB drop in the SPL. Moreover, the residual stress, as well as the stress gradient present in the deposited composite stacking layers, deforms the cantilever beam, thus widening the air gap further, especially around the tip region. Therefore, the effect of the residual stress and its gradient on device performance is also studied. Figure 2d shows the changes in the air gap between adjacent cantilever tips and the corresponding SPL at 20 Hz under different residual stresses. With increasing residual stress, the air gap becomes increasingly wider, followed by a monotonic decrease in the SPL. For a residual stress of 320 MPa in the as-deposited stacking layer, the tip air gap increases from 6.8 µm to 8.2 µm, resulting in an additional SPL reduction of nearly 0.86 dB.

**Fig. 3:**
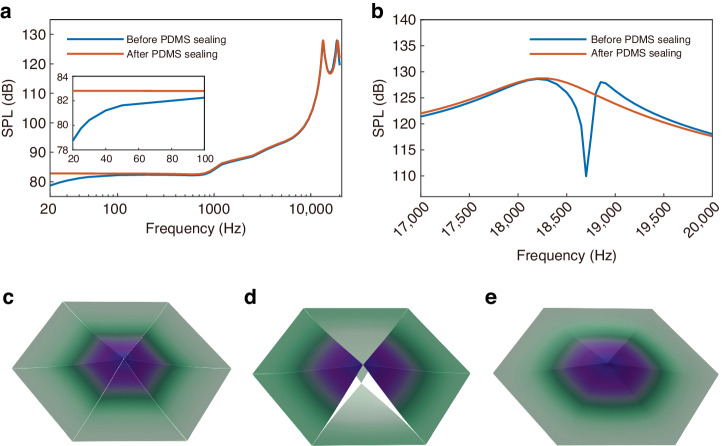
**Simulation results of piezoelectric MEMS speaker before and after gap sealing. a** SPL curves of the speaker. **b** SPL curves around the resonant frequency of the speaker, where the lengths of four beams differ from the others $(\Delta{L} = 20\, {\mu}{m})$. **c** First resonance mode of the speaker with six identical cantilever beams, f = 18.8 kHz. **d** Asynchronous vibration mode of the speaker with four extra cantilever beams added, f_1_ = 18.4 kHz, f_2_ = 18.8 kHz. **e** Modal characteristics of the sealed speaker with PDMS, f = 18.2 kHz

The SPL frequency responses generated by the speaker in the audio range from 20 Hz to 20 kHz at a sinusoidal driving voltage of 40 V_pp_ are studied before and after sealing treatment with PDMS, as shown in Fig. 3a. For clarity, the SPL curve from 20 Hz to 100 Hz is magnified and presented as an inset. Sealing clearly improves the SPL in the low-frequency range; the SPL improved to 4.07 dB at a 20 Hz operation frequency. In contrast, the SPL response in the middle- and high-frequency regions remains nearly unchanged, which is mainly attributed to the lower material strength of the PDMS and its selective sealing ability.

Moreover, the issue induced by the inconsistent mechanical characteristics of cantilever beams due to the fabrication process is also studied. Figure 3c shows the resonant vibration mode of the speaker at 19 kHz in the ideal case in which all of the cantilever beams are identical, resulting in synchronized vibration. To simulate the process-induced dimension change, four of the cantilever beams were intentionally designed to be 20 μm longer than the other two. In this case, the speaker demonstrated two closely spaced resonant frequencies, namely, 18.4 kHz and 18.8 kHz. When all of the cantilever beams are driven using the same control signal of 40 V_pp_ and the frequency falls between these two resonant frequencies, a distinct asynchronous vibration between the cantilever beams occurs, as shown in Fig. 3d. This vibration induces a significant decrease in the SPL response of more than 20 dB (see Fig. 3b). After the air gap is sealed with PDMS, all of the cantilever beams are mechanically coupled as a new combination with a resonant frequency of 18.2 kHz, forcing them into synchronous vibration, as shown in Fig. 3e. As a result, the abovementioned decrease in the SPL is completely eliminated, and the SPL is improved to approximately 16.5 dB at 18.7 kHz.

**Fig. 4:**
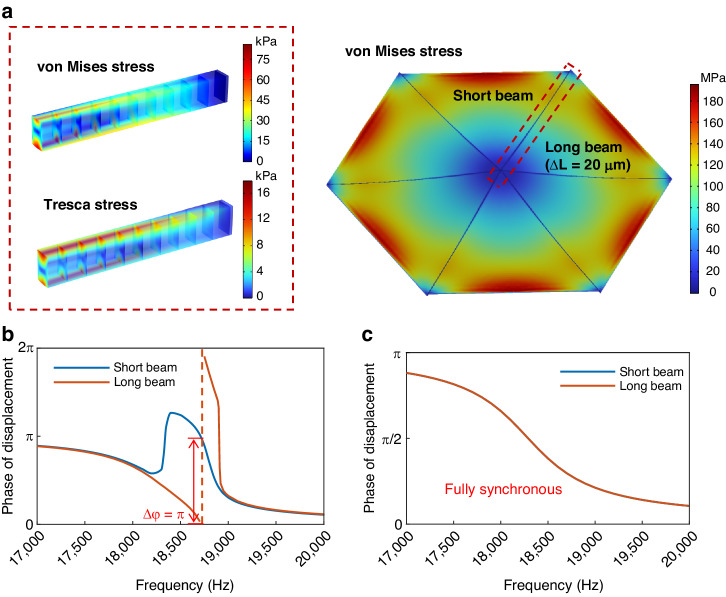
**Stress distribution in the speaker and phase frequency spectrum curves. a** Stress distribution in the speaker at the resonant frequency, the inserts illustrate the von Mises stress and the Tresca stress within the PDMS in the gap, respectively. **b** Phase of displacement at the tips of the short and long beams before sealing. **c** Phase of displacement at the tips of the short and long beams after sealing

The phase variations in the tip displacement of the short and long beams are shown in Fig. 4b, c. Due to the different resonant frequencies and the effect of acoustic crosstalk, the initial phase variations near the resonant frequency of these two sets of cantilever beams significantly differ, which represents asynchronous movement. The phases of these two sets of beams begin to diverge after 18.2 kHz, and the maximum phase difference of ${\rm{\pi }}$ occurs at 18.7 kHz, which is also the frequency point at which the decrease in SPL reaches a maximum. Subsequently, the phase difference gradually decreases and returns to synchronous vibration. After PDMS sealing, the cantilever beams maintain complete phase alignment near the resonant frequency, indicating that these two sets of beams are consistently connected by PDMS, forming a unified structure. The PDMS inside the gap serves as a connecting spring, effectively overcoming the relative motion between adjacent cantilever beams and improving asynchronous vibration phenomena.

To assess the operational reliability of the speaker after sealing, the stress distribution in the speaker is extracted for a resonant frequency of 18.2 kHz at 40 V_pp_, as shown in Fig. 4a. Due to the vibration amplification effect under resonance, the displacement of the cantilever beams in the MEMS speaker is maximized, which results in a von Mises stress peak of 196 MPa in the cantilever beams. Moreover, increasing the displacement of the cantilever beam toward its tip amplifies the tension exerted on the PDMS, and stress in the PDMS is maximized around the tip region. The maximum von Mises stress and maximum Tresca stress within the PDMS are 88.1 kPa and 17.9 kPa, respectively. The tensile strength and shear strength of the PDMS are 6.9 MPa and 0.38 MPa^30^, respectively, far exceeding the maximum von Mises stress and maximum Tresca stress within the PDMS. As a result, fracture within the PDMS inside the gap is unlikely to occur, confirming the reliability of the sealing method.

### Fabrication

**Fig. 5:**
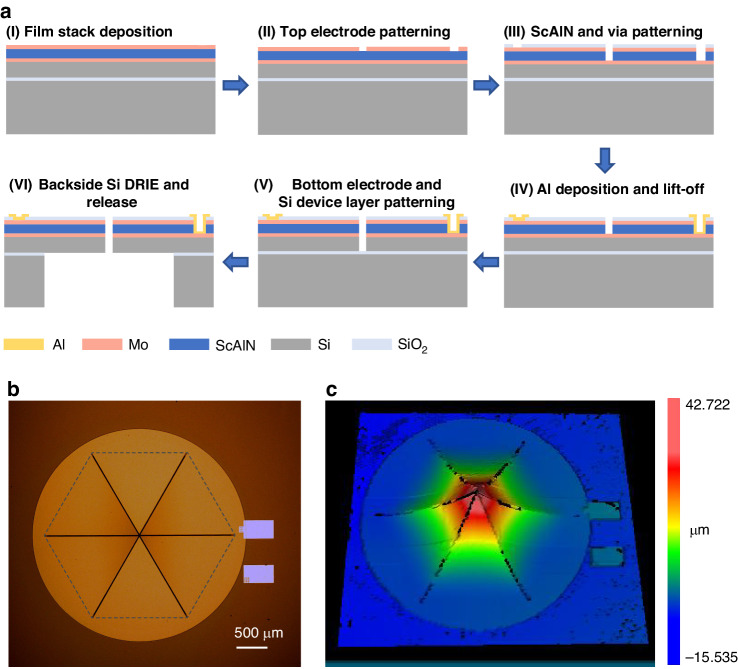
**Fabrication and optical morphology characterization of the proposed piezoelectric MEMS speaker**. **a** Process flowchart of the fabrication. **b** Microscope image. **c** Three-dimensional profile obtained by white light interferometry

The fabrication process of the proposed MEMS speaker is illustrated in Fig. 5a. The process starts with an 8-inch silicon-on-insulator (SOI) wafer including a 10 μm thick Si (Silicon) device layer and a 0.5 μm thick buried oxide layer. Initially, a series of depositions were carried out on the SOI wafer via the magnetron sputtering method, involving 0.2 μm of bottom electrode Mo (Molybdenum), 1 μm of piezoelectric ScAlN (Scandium-doped Aluminum Nitride, with a Sc content of 9.6%) and an additional 0.2 μm of top electrode Mo. Subsequently, dry etching was employed to pattern the top electrode layer. These steps were followed by the patterning of the piezoelectric layer, growth of the passivation layer, and opening to the top and bottom electrodes. The next step involved the fabrication of patterned Al (Aluminum) electrodes with a thickness of 1 μm via a lift-off process, which connect to the top and bottom electrodes of the speaker. Both the bottom electrode and the Si device layer were then selectively etched to form cantilever beams and air gaps. Finally, the Silicon of the back cavity was deeply etched and the buried oxide layer was released. The material was then diced to generate individual speakers.

## Results

The morphology of the as-fabricated speaker was characterized using an optical microscope and a white-light interferometer (Zygo-NewView 7300, U.S.A.), as shown in Fig. 5. The measurement of the speaker’s three-dimensional contour clearly showed the upward bending of the cantilever beams, with a measured tip deflection of 42.14 μm, indicating the presence of a significant residual tensile stress of approximately 320 MPa in the as-deposited stack layer. Moreover, the bending of the cantilever increased the air gap width from 6.8 μm around the clamped region to 8.3 μm at the tip region for the tested device, which is consistent with the simulation results. Notably, the final air gap width between the cantilevers is related to their initial deflections, which depend on the residual stress in the as-deposited stack layers. Because a certain stress distribution exists in the as-deposited material across the entire wafer area, the final air gap width in different devices on the same wafer might slightly differ.

**Fig. 6:**
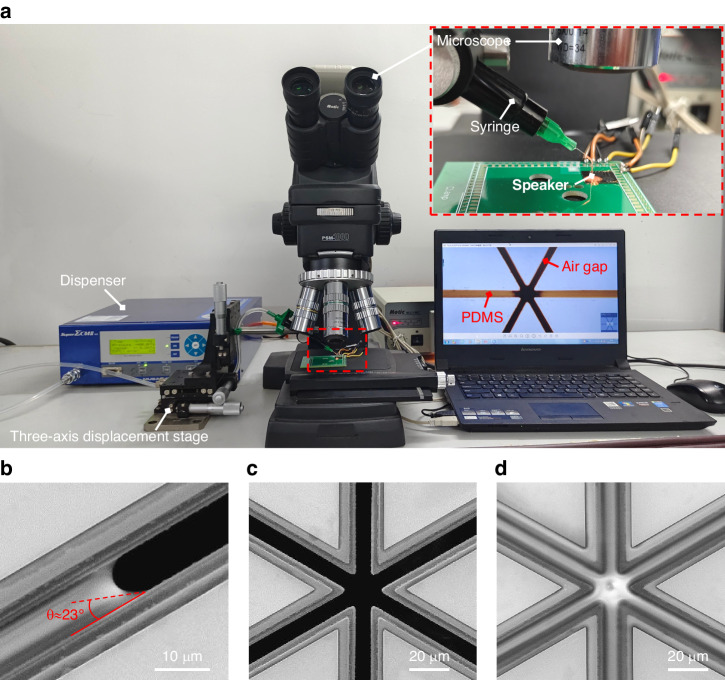
**Speaker gap sealing process and SEM images. a** Gap sealing platform. **b** Contact angle measurement. **c** The tip region before sealing. **d** The tip region after sealing

A speaker gap sealing operation platform was constructed using a three-axis displacement stage and a microscope, as shown in Fig. 6a. First, the liquid PDMS prepolymer (a mixture of the PDMS base and the curing agent at a mass ratio of 10:1) was prepared and loaded into a syringe. The syringe was then clamped onto the three-axis displacement stage, allowing precise positioning with the assistance of the microscope. Finally, a dispenser (MUSASHI-SUPER ΣCMII V5, Japan) was used to place a liquid PDMS droplet at the edge region of the air gaps, which are intentionally extended outside the cantilever beams on the substrate of the MEMS speaker. A syringe equipped with a 34 G needle was used in the experiment, and the volume of the dispensed liquid PDMS was controlled by adjusting the air pressure and the dispensing time to prevent excessive PDMS from spreading onto undesired cantilever regions. Although PDMS has high transmittance for visible light ( > 93%), it can still be observed under a microscope. In Fig. 6a, the liquid PDMS is visible as a dark yellow color in the gap region, and the unsealed gaps remain black under the microscope. During operation, the PDMS flowed along the air gap from its edge to its center due to the capillary effect. When all of the air gaps were sealed, the device was placed in an oven to cure the filled liquid PDMS.

Theoretical analysis revealed that the liquid PDMS can automatically flow in the air gap formed between the Si sidewalls once the spontaneous capillary flow (SCF) condition is met^31^:

14 $$\frac{G}{H} < \cos (\theta )$$

where $G$ represents the width of the gap, $H$ represents the thickness of the Si device layer and $\theta$ represents the contact angle between the liquid PDMS and the Si sidewall surface.

To better reveal the filling procedure, SEM images of the speaker are also provided, as shown in Fig. 6b. Figure 6c, d, all of which were captured before curing. As shown in Fig. 6b, the contact angle between the liquid PDMS and the Si sidewall surface was measured to be approximately 23°. Considering the 10 μm thick Si layer in the current design, the gap width should not exceed 9.21 μm to achieve SCF. The above measurement results indicate that the maximum gap width of 8.3 μm in the as-fabricated piezoelectric MEMS speaker occurs at the cantilever beam tip region. As a result, the liquid PDMS completely fills all gaps in the device, as validated by the experimental results shown in Fig. 6d. In addition, PDMS did not accumulate or overflow, which also demonstrates the self-constrained flow characteristics of the liquid PDMS in the gap. Therefore, the sealing method based on the capillary effect is easy to control.

**Fig. 7:**
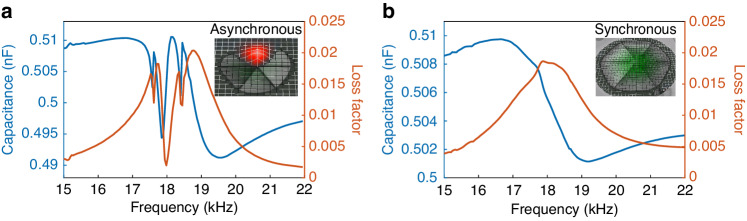
**LCR and mode shape measurement results before and after sealing. a** Before sealing. **b** After sealing

Various measurements were obtained before and after air gap sealing to evaluate the electrical, mechanical and acoustic characteristics of the speaker. First, the capacitance and loss factor frequency spectra of the device were measured using an LCR meter (Agilent-E4980A, USA), as depicted in Fig. 7. Before PDMS sealing, the capacitance and loss factor curves of the speaker distinctly fluctuated in the frequency range from 17 kHz to 19 kHz (see Fig. 7a). Due to the inconsistent dimensions of the six cantilever beams induced by fabrication, their resonant frequencies are also different, resulting in different capacitance and loss curves for each cantilever beam. Because the electrodes of all six cantilever beams are connected in parallel, the measured results are in fact the superposition of all six cantilever beams, resulting in multipeaked characteristics in the capacitance and loss curves. The inset of Fig. 7a also shows the mode shapes of the speaker measured by a laser Doppler vibrometer (Polytec-MSA 600, Germany), which clearly shows the asynchronous vibration between the cantilever beams, further confirming the existence of a resonant frequency discrepancy. In comparison, after the sealing treatment, the six cantilever beams are connected as a whole with a single resonant frequency; hence, multiple resonance peaks are merged into one in the capacitance and loss curves. The LCR curves indicate that all of the cantilever beams are currently in synchronous working mode, which is also supported by the laser Doppler velocimetry measurement results provided in the inset of Fig. 7b, thus validating the sealing effectiveness of the simulation.

**Fig. 8:**
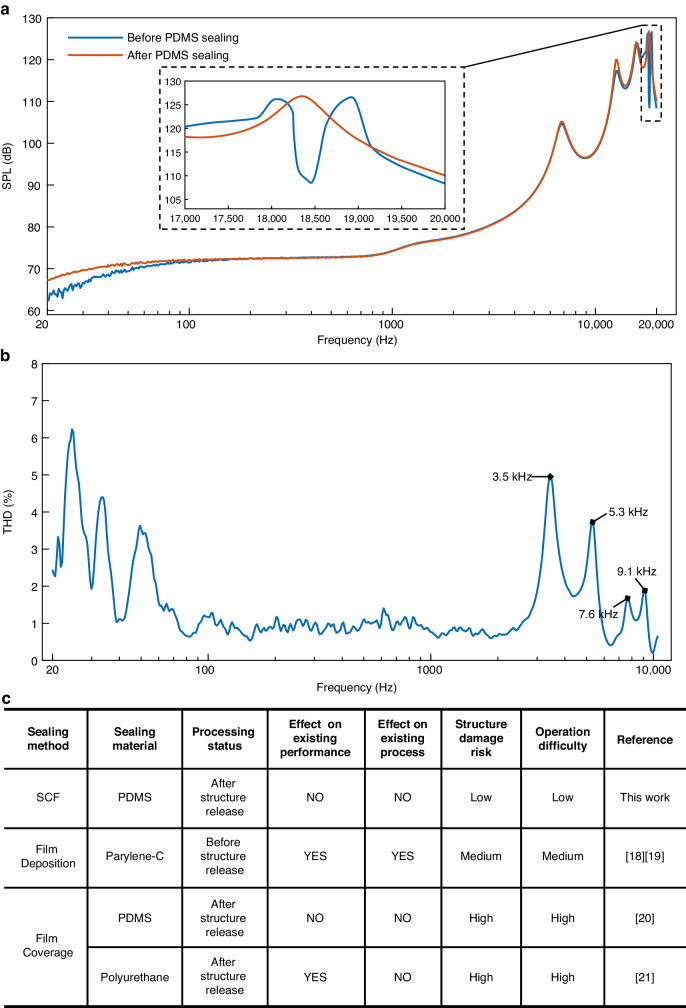
**Acoustic measurement results of the piezoelectric MEMS speaker before and after sealing.** **a** SPL frequency spectrum curves. **b** THD frequency spectrum curves. **c** Comparison about sealing methods of state-of-the-art

The as-fabricated piezoelectric MEMS speaker was acoustically characterized in an IEC ear simulator (B&K-4195, Denmark), and the results are shown in Fig. 8. During the measurement, the speaker was excited by a 40 V_pp_ sinusoidal chirp signal with a frequency ranging from 20 Hz to 20 kHz. After the air gaps were sealed by PDMS, the SPL markedly improved in the frequency range lower than 100 Hz. Specifically, the SPL improved to 4.9 dB at 20 Hz, which is attributed to the prevention of the acoustic short circuit by the sealing treatment.

In addition, before sealing, the SPL frequency spectrum of the speaker displays a distinct decrease in amplitude between 18 kHz and 19 kHz, as shown in the insert of Fig. 8a. This decrease is a result of the asynchronous vibration between the cantilever beams that leads to the mutual cancellation of sound signals, which is in good agreement with the simulation results. In comparison, sealing effectively prevents asynchronous vibration, and all of the cantilever beams are forced to vibrate in synchronous mode as a whole combination structure. As a result, the SPL significantly improved to 17.5 dB in the decreased region. Moreover, the SPL response in the frequency range from 100 Hz to 16 kHz remained nearly constant after sealing, as expected, and negligible changes in the acoustic characteristics of the sealed speaker can be observed after extended operation, demonstrating the excellent robustness of the sealing method.

The THD frequency spectrum curves of the as-fabricated piezoelectric MEMS speaker are shown in Fig. 8b. The speaker exhibits relatively a high THD below 100 Hz, which is primarily attributed to environmental noise and electromagnetic interference, which also leads to fluctuations in the SPL curve shown in Fig. 8a. Incomplete noise isolation contributes to higher harmonic components and results in an overestimation of THD measurements. Due to the relatively low SPL of the speaker, the impact of noise is more significant, leading to pronounced fluctuations in the measured THD from 100 Hz to 1 kHz. The THD of the speaker was measured to be 0.728% at 1 kHz for a 40 V_pp_ driving voltage. At higher frequencies, the THD curve smoothens because of the reduction in environmental noise and the increase in the SPL. Due to the subharmonic oscillation, the THD curve exhibits peaks at 3.5 kHz, 5.3 kHz, 7.6 kHz and 9.1 kHz, which are half or a third of the resonant frequencies of the IEC ear simulator and the speaker^17,32^. From 100 Hz to 10 kHz, the THD of the proposed speaker remained below 5%, further demonstrating good linearity.

Moreover, cured PDMS can operate reliably over a temperature range of −45 to 200 °C for long periods of time^33^. In our current experiment, a negligible change in the performance of the MEMS speaker after PDMS sealing was observed after continuous operation for several days.

## Discussion and conclusion

Among existing piezoelectric MEMS speaker technology, cantilever beam-like actuator designs have attracted more extensive attention due to their distinct advantages, such as high SPL output capability, lower sensitivity to the fabrication process-related residual stress and large linear operation range. However, the inevitable air leakage associated with the air gap in the structure will lead to a noticeable SPL decrease in the low-frequency range. In addition, the asynchronous vibration phenomenon, which is caused by process-induced inconsistencies in the mechanical performance between multiple cantilever beams, significantly deteriorates the SPL near the resonant frequency further.

In this work, the air leakage and asynchronous vibration phenomena of the proposed piezoelectric MEMS speaker were validated through modeling and experimental measurements. FEM simulations reveal that the SPL of the MEMS speaker decreases with increasing air gap width. Due to the initial deformation of the cantilever beams caused by the residual stress, the air gap widened further, especially around the tip region, leading to an additional decrease in the SPL. Moreover, asynchronous vibration occurred near the resonant frequencies when the six cantilever beams were not perfectly matched, causing a significant reduction in the SPL.

To address these problems, a novel sealing strategy based on the capillary effect was proposed. Both the simulation results and experimental measurements confirmed the suppression of air leakage and asynchronous vibration after PDMS sealing. The LCR measurement results indicate multiple discrete resonance peaks in the prepared piezoelectric MEMS speaker, which merge into a single peak after the sealing operation. Acoustic measurements were conducted using an IEC human ear simulator, the results of which indicate that the SPL increased by 4.9 dB at 20 Hz after sealing. An improved SPL of approximately 17.5 dB was successfully achieved near the resonant frequency of the cantilever beams. A comparison of the gap sealing method used in this work and the state-of-the-art methods is provided in Fig. 8c. The proposed sealing approach offers advantages, including convenient operation, precise control and minimal impact on mechanical characteristics. Notably, this strategy is also applicable to other air-coupled MEMS acoustic devices that feature air gap structures and encounter air leakage issues, such as MEMS microphones and PMUTs. Furthermore, as a polymer material, PDMS also faces aging issues induced by UV exposure^34^. Therefore, if the MEMS speaker operates in an environment with significant UV light exposure, appropriate packaging should be designed for protection purposes.

In the future, the optimization of piezoelectric MEMS speaker designs is expected to further improve the SPL in the low-frequency range. Other microfluidic structures may also be involved in the device design to improve the precision and ease of operation of the proposed sealing strategy.

## References

[1] Wang, H. et al. Review of Recent Development of MEMS Speakers. *Micromachines* **12**, 1257 (2021). 34683308 10.3390/mi12101257 PMC8537663

[2] Shahosseini, I. et al. Optimization and Microfabrication of High Performance Silicon-Based MEMS Microspeaker. *IEEE Sens. J.* **13**, 273–284 (2013). 10.1109/JSEN.2012.2213807

[3] Setiarini, A. et al. A Novel Structure of Electromagnetic MEMS Speaker for Hearing Aid Application. in *2018 International Conference on Radar, Antenna, Microwave, Electronics, and Telecommunications (ICRAMET)* 112–116 (IEEE, 2018).

[4] Shahosseini, I. et al. Electromagnetic MEMS microspeaker for portable electronic devices. *Microsyst. Technol.* **19**, 879–886 (2013). 10.1007/s00542-013-1754-7

[5] Kaiser, B. et al. Concept and proof for an all-silicon MEMS micro speaker utilizing air chambers. *Microsyst. Nanoeng.* **5**, 1–11 (2019). 31636932 10.1038/s41378-019-0095-9 PMC6799830

[6] Roberts, R. C. et al. Electrostatically Driven Touch-Mode Poly-SiC Microspeaker. in *2007 IEEE Sensors* 284–287 (IEEE, 2007).

[7] Chen, Y.-C. et al. On the design of a two-way piezoelectric MEMS microspeaker based on a multi-shape cantilever array for high-frequency applications. *J. Micromech. Microeng.* **33**, 074001 (2023). 10.1088/1361-6439/acceb1

[8] Sun, M. et al. Broadband MEMS Speaker by Single-Way Multi-Resonance Array with Acoustic Damping Tuning: A Proof of Concept. in *2023 IEEE 36th Internation*al *Conference on Micro Electro Mechanical Systems (MEMS)* 677–680 (IEEE, 2023).

[9] Shao, Z. et al. A Single Chip Directional Loudspeaker Based on PMUTS. in *2021 IEEE 34th International Conference on Micro Electro Mechanical Systems (MEMS)* 895–898 (IEEE, 2021).

[10] Wang, H., Feng, PX-L. & Xie, H. A Dual-Electrode MEMS Speaker Based on Ceramic PZT with Improved Sound Pressure Level by Phase Tuning. in *2021 IEEE 34th International Conference on Micro Electro Mechanical Systems (MEMS)* 701–704 (IEEE, 2021).

[11] Wang, Q. et al. A Piezoelectric MEMS Speaker with a Combined Function of a Silent Alarm. *Micromachines* **14**, 702 (2023). 36985109 10.3390/mi14030702 PMC10057705

[12] Cheng, H.-H. et al. Monolithic Integration of PZT Actuation Units of Various Activated Resonances for Full-Range MEMS Speaker Array. in *2023 IEEE 36th International Conference on Micro Electro Mechanical Systems (MEMS)* 685–688 (IEEE, 2023).

[13] Gazzola, C. et al. On the Design and Modeling of a Full-Range Piezoelectric MEMS Loudspeaker for In-Ear Applications. *J. Microelectromech. Syst.* **32**, 626–637 (2023). 10.1109/JMEMS.2023.3312254

[14] Stoppel, F. et al. Novel membrane-less two-way MEMS loudspeaker based on piezoelectric dual-concentric actuators. in *2017 19th International Conference on Solid-State Sensors, Actuators and Microsystems (TRANSDUCERS)* 2047–2050 (IEEE, 2017).

[15] Yuan, N.-H. et al. Acoustic Performance of Stress Gradient-Induced Deflection of Triangular Unimorphic/Bimorphic Cantilevers for MEMS Applications. *Materials* **16**, 2129 (2023). 36903244 10.3390/ma16052129 PMC10004114

[16] Fawzy, A. Membraneless Piezoelectric MEMS speakers based on AlN Thin Film. *JES J. Eng. Sci.* **50**, 1–8 (2022).

[17] Liechti, R. et al. High performance piezoelectric MEMS loudspeaker based on an innovative wafer bonding process. *Sens. Actuator A-Phys.* **358**, 114413 (2023). 10.1016/j.sna.2023.114413

[18] Ma, Y. et al. A PZT MEMS loudspeaker with a quasi-closed diaphragm. *Sens. Actuator A-Phys.* **358**, 114454 (2023). 10.1016/j.sna.2023.114454

[19] Wang, Q. et al. Obtaining High SPL Piezoelectric MEMS Speaker via a Rigid-Flexible Vibration Coupling Mechanism. *J. Microelectromech. Syst.* **30**, 725–732 (2021). 10.1109/JMEMS.2021.3087718

[20] Xu, L. et al. A Piezoelectric MEMS Speaker with Stretchable Film Sealing. in *2023 IEEE 36th International Conference on Micro Electro Mechanical Systems (MEMS)* 673–676 (IEEE, 2023).

[21] Kuchiji, H., Masumoto, N. & Baba, A. Piezoelectric MEMS wideband acoustic sensor coated by organic film. *Jpn. J. Appl. Phys.* **62**, SG1021 (2023). 10.35848/1347-4065/acbb82

[22] Berthier, J., Brakke, K. A. & Berthier, E. A general condition for spontaneous capillary flow in uniform cross-section microchannels. *Microfluid. Nanofluid.* **16**, 779–785 (2014). 10.1007/s10404-013-1270-1

[23] Ledesma, E., Zamora, I., Uranga, A. & Barniol, N. 9.5% Scandium Doped ALN PMUT Compatible with Pre-Processed CMOS Substrates. in *2021 IEEE 34th International Conference on Micro Electro Mechanical Systems (MEMS)* 887–890 (IEEE, 2021).

[24] Caro, M. A. Piezoelectric coefficients and spontaneous polarization of ScAlN. *J. Phys.* **27**, 245901 (2015). 10.1088/0953-8984/27/24/245901 26000892

[25] Berthier, J. et al. The Dynamics of Spontaneous Capillary Flow in Confined and Open Microchannels. *Sens. Transducers* **183**, 123 (2014).

[26] Hong, Y. et al. Theoretical analysis and experimental study of the effect of the neutral plane of a composite piezoelectric cantilever. *Energ. Convers. Manag.* **171**, 1020–1029 (2018). 10.1016/j.enconman.2018.06.045

[27] Tzou, H. S. & Gadre, M. Theoretical analysis of a multi-layered thin shell coupled with piezoelectric shell actuators for distributed vibration controls. *J. Sound Vib.* **132**, 433–450 (1989). 10.1016/0022-460X(89)90637-8

[28] Hosseini, R. & Hamedi, M. Study of the Resonant Frequency of Unimorph Triangular V-shaped Piezoelectric Cantilever Energy Harvester. *Int. J. Ad. Manuf. Tec.* **8**, 75–82 (2015).

[29] IEC 60318-4: Electroacoustics simulators of human head and ear part 4: occluded-ear simulator for the measurement of earphones coupled to the ear by means of ear inserts (Switzerland, 2010).

[30] Wang, Y. et al. Enhanced mechanical and adhesive properties of PDMS based on novel PDMS-epoxy IPN structure. *J. Polym. Res.* **28**, 171 (2021). 10.1007/s10965-021-02518-w

[31] Berthier, J. et al. Suspended microflows between vertical parallel walls. *Microfluid. Nanofluid.* **18**, 919–929 (2015). 10.1007/s10404-014-1482-z

[32] Hirano, Y. et al. PZT MEMS Speaker Integrated with Silicon-Parylene Composite Corrugated Diaphragm. in *2022 IEEE 35th International Conference on Micro Electro Mechanical Systems Conference (MEMS)* 255–258 (IEEE, 2022).

[33] Sylgard 184 silicone elastomer (2024). at https://www.dow.com/zh-cn/document-viewer.html #docPath=/content/dam/dcc/documents/11/11-3184-01-sylgard-184-elastomer.pdf.

[34] Charitidis, C. A. et al. Influence of accelerated aging on nanomechanical properties, creep behaviour and adhesive forces of PDMS. *Plast. Rubber Compos.* **41**, 94–99 (2012). 10.1179/1743289811Y.0000000013

[35] Material: parylene (2024). at https://www.mit.edu/~6.777/matprops/parylene.htm.

[36] Material: Polyimide (2024). at https://www.mit.edu/~6.777/matprops/polyimide.htm.

